# Increased HOXC6 mRNA expression is a novel biomarker of gastric cancer

**DOI:** 10.1371/journal.pone.0236811

**Published:** 2020-08-03

**Authors:** Jiyoon Jung, Sanghoon Jeong, Hoiseon Jeong, Hwa Eun Oh, Jung-Woo Choi, Eung Seok Lee, Young-Sik Kim, Yoonjin Kwak, Woo Ho Kim, Ju-Han Lee

**Affiliations:** 1 Department of Pathology, Ansan Hospital, Korea University College of Medicine, Ansan-Si, Gyeonggi-Do, Republic of Korea; 2 Medical Science Research Center, Ansan Hospital, Korea University College of Medicine, Ansan-si, Gyeonggi-Do, Republic of Korea; 3 Department of Pathology, Seoul National University Hospital, Seoul, Republic of Korea; Istituto di Ricovero e Cura a Carattere Scientifico Centro di Riferimento Oncologico della Basilicata, ITALY

## Abstract

In this study, we aimed to investigate the molecular biomarkers that are pivotal for the development and progression of gastric cancer (GC). We analyzed clinical specimens using RNA sequencing to identify the target genes. We found that the expression of *HOXC6* mRNA was upregulated with the progression of cancer, which was validated by quantitative real time PCR and RNA *in-situ* hybridization. To compare the protein expression of HOXC6, we evaluated GC and normal gastric tissue samples using western blot analysis and immunohistochemistry. We detected significantly higher levels of HOXC6 in the GC tissues than in the normal controls at both mRNA and protein levels. The expression levels of *HOXC6* mRNA in patients with advanced gastric cancer (AGC) were significantly higher than those in patients with early gastric cancer (EGC). Kaplan-Meier curves showed that high expression of *HOXC6* mRNA is significantly associated with poor clinical prognosis. Our findings suggest that *HOXC6* mRNA may be a novel biomarker and can be potentially valuable in predicting the prognosis of GC patients. Especially, *HOXC6* mRNA *in-situ* hybridization may be a diagnostic tool for predicting prognosis of individual GC patients.

## Introduction

Gastric cancer (GC) is the fifth most common cancer and the third leading cause of cancer death worldwide with markedly higher incidence rates in East Asian countries [1]. In Korea, use of endoscopy to screen for GC has facilitated early detection and improved survival. The proportion of GC patients in the screening population increased to 65.4%, and the proportion of patients with stage I cancer among the entire patient population also increased to 70.6% by the year 2011 [2]. Endoscopic submucosal dissection (ESD) has become a standard treatment strategy for selected cases of early gastric cancer (EGC). The prognosis of EGC is excellent with an overall 5-year survival rate of 96.6% and disease specific-free survival rate of 90.6% [3]. However, the 5-year survival rate of advanced gastric cancer (AGC) with perigastric lymph node metastasis is 37.9% [4]. Thus, it is particularly important to detect lymph node metastasis before treatment.

Owing to the differences in the therapeutic options and survival rates, it is crucial to determine the molecular biomarkers that are pivotal for the development and progression of GC. Moreover, the identification of biomarkers, followed by the development of targeted therapies, may improve the clinical outcomes [5]. Recently, RNA sequencing technology has emerged as a powerful method for screening transcripts. The expression profiles of the genes involved in GC have been extensively investigated, yielding useful insights into the molecular mechanism of GC [6].

To discover the specific biomarkers for GC with specific focus on lymph node metastasis, we investigated the differentially expressed genes (DEGs) between GC tissues (GC patients with and without lymph node metastasis) and corresponding normal tissue using RNA sequencing. Our results suggested that the expression of *HOXC6* mRNA might be associated with gastric carcinogenesis. Subsequently, we used various methods to validate *HOXC6* mRNA as a biomarker for GC. In addition, we investigated the relationship between the clinicopathologic characteristics of GC and HOXC6 expression at the mRNA and protein levels.

## Materials and methods

### RNA sequencing of tissue samples

Between February 2016 and November 2016, six patients with GC (three AGC patients without lymph node metastasis and three AGC patients with lymph node metastasis) were included in the current study; their diagnoses were pathologically confirmed as adenocarcinoma. The GC tissues and paired normal gastric tissues were obtained from surgical specimens. Fresh GC and matched normal gastric tissue samples were provided by the Biobank of Korea University Ansan Hospital. The use of tissue samples was approved by the Institutional Review Board of Ansan Medical center (IRB no: 2018AS0092)

### RNA extraction, library preparation, and sequencing

Total RNA was isolated using TRIzol reagent (Invitrogen), and the quality of the extracted RNA was assessed using an Agilent 2100 bioanalyzer using the RNA 6000 Nano Chip (Agilent Technologies, Amstelveen, The Netherlands). RNA quantification was performed using an ND-2000 Spectrophotometer (Thermo Inc., DE, USA).

For generating the control and test RNA samples, an RNA library was constructed using QuantSeq 3’ mRNA-Seq Library Prep Kit (Lexogen, Inc., Austria) according to the manufacturer’s instructions. Briefly, 500 ng of each total RNA sample was prepared and an oligo-dT primer containing an Illumina-compatible sequence at its 5’ end was hybridized to the RNA followed by reverse transcription. After degradation of the RNA template, second strand synthesis was initiated using a random primer containing an Illumina-compatible linker sequence at its 5’ end. The double-stranded library was purified using magnetic beads to remove all the reaction components. The library was amplified to add the complete adapter sequences required for cluster generation and after amplification, it was purified from the PCR components. High-throughput sequencing was performed as single-end 75 sequencing using NextSeq 500 (Illumina, Inc., USA).

### Data analysis

QuantSeq 3’ mRNA-Seq reads were aligned using Bowtie2 [7]. Bowtie2 indices were either generated from the genome assembly sequence or the representative transcript sequences for aligning to the genome and transcriptome. The alignment file was used for assembling transcripts, estimating their abundances, and detecting DEGs. DEGs were determined based on the counts from unique and multiple alignments using Bedtools coverage tool [8]. The Read count data was processed based on Quantile normalization method using EdgeR within R using Bioconductor [9]. Gene classification was done by conducting searches on DAVID (http://david.abcc.ncifcrf.gov/) and Medline databases (http://www.ncbi.nlm.nih.gov/).

### Oncomine database analysis

To analyse the expression level of *HOXC6* in GC, Oncomine [https://www.oncomine.org, Compendia biosciences, Ann Arbor, MI, USA] [10], an online microarray database was used. The mRNA expression fold in cancer tissue compared to the normal tissue was obtained as the parameters of p-value<1E-4, fold change>2, and gene ranking in the top 10%. The p value, and fold changes were extracted. The eligible dataset was generated by Human Genome U133 Plus 2.0 Array [11].

### Real time PCR

Fresh GC and normal gastric tissue samples were obtained from the Biobank of Korea University Ansan Hospital and Keimyung University Dongsan Hospital. A total of 13 normal gastric tissue, 8 EGC tissue, and 52 AGC tissue (18 cases without lymph node metastasis and 34 cases with lymph node metastasis) samples were obtained. Total RNA was extracted from each tissue sample using TRIzol reagent (Invitrogen, Carlsbad, CA, USA). Using the total RNA, reverse transcription was performed using SuperScript II Reverse Transcriptase (Invitrogen, Carlsbad, CA, USA) according to the manufacturer’s instructions. cDNA was amplified from the mRNA using the primer pairs as follows: *HOXC6* (forward: 5’- TGACCGTTTCTGTGTGAAGA -3’, reverse: 5’- AGGAACACTGACGGTGCTAA-3’), β-actin (forward: 5’- AATGCTTCTAGGCGGACTATGA -3’, reverse: 5’- TTTCTGCGCAAGTTAGGTTTT -3’). Real time PCR was performed on the StepOnePlusTM Real Time PCR System (Applied Biosystems, USA) using SYBR Green PCR Kit (Applied Biosystems, USA), according to the manufacturer’s instructions. The thermal cycling conditions were 95°C for 10 min followed by 40 cycles at 95°C for 15 s and finally, 30 s at optimal T_m_ (59°C). The data were analyzed using the StepOne software v2.2.2 (Applied Biosystems, USA). The expression levels of each mRNA was normalized to the expression of endogenous β-actin as control and the values were calculated using the 2-ΔΔCt method.

### RNA *in-situ* hybridization

The specimens were collected from the patients who underwent gastrectomy at the Korea University Ansan Hospital between 2018 and 2019. In total, 8 normal gastric tissue, 15 EGC tissue, and 28 AGC tissue (12 AGC tissues without lymph node metastasis and 16 AGC tissues with lymph node metastasis) samples were obtained and used to prepare formalin-fixed paraffin-embedded (FFPE) sections.

RNA *in*-*situ* hybridization experiments were performed using RNAscope®, an RNA *in-situ* hybridization method described previously [12]. Paired double-Z oligonucleotide probes were designed against the target RNA using a custom software. The following probes were used (*HOXC6*, cat no. 312211, NM_153693, 20 pairs, and probe target region 575–2032). The RNAscope Intro Pack 2.5 HD Reagent Kit (Advanced Cell Diagnostics, Newark, CA) was used, and the FFPE tissue sections were prepared according to manufacturer’s instructions. Each sample was subjected to quality control to check for RNA integrity with a probe specific to the housekeeping gene PPIB (peptidylprolyl isomerase B). The negative control background staining was evaluated using a probe specific to the bacterial dapB gene. Bright field images were acquired using an OLYMPUS BX51 microscope with a 1000x objective.

### Kaplan-Meier (KM) plot

The prognostic value of *HOXC6* mRNA transcription level was measured using KM plotter, an online open database (www.kmplot.com), which consists of gene expression profiles and survival information of GC patients. Using this database, the level of *HOXC6* mRNA transcription was measured using the HGU133A platform. A total of 320 intestinal type GC patients were used for the analysis. These patients were divided into two groups based on the expression of *HOXC6*. Patients with higher *HOXC6* expression than the median were separately pooled into the group with high expression, while those with lower *HOXC6* expression than the median were pooled into the group with low expression. Other statistical outcomes, including hazard ratio (HR), 95% confidence intervals (CI), and log rank P were also calculated using this database. P values < 0.05 were used to indicate statistically significant difference.

### Western blot

Western blot analysis was used to evaluate the protein expression levels of HOXC6 in 10 normal gastric tissue and 10 AGC tissue samples. The samples were lysed using T-PER™ Tissue Protein Extraction Reagent (Thermo Fisher Scientific Inc., Rockford, IL, USA) and the protein concentration was measured. The protein extracts (25μg) were separated by 15% sodium dodecyl sulfate polyacrylamide gel electrophoresis and transferred onto polyvinylidene fluoride membrane. The blots were blocked with 5% skim milk at room temperature for 60 min and then incubated with HOXC6 (1:1000, sc-376330) and GAPDH (1:4000, sc-47724) primary antibody obtained from Santa Cruz Biotechnology (Santa Cruz, CA, USA) at 4°C overnight. After washing thrice (10 min each time) with Tris-buffered saline with Tween 20, the membranes were incubated with the goat anti-mouse secondary antibody (1:2000, 7076S, Cell Signaling Technology, Beverly, MA, USA) for 60 min and rinsed as described before. We applied the chemiluminescent substrate (GenDEPOT, Baker, TX, USA) to the membrane and detected the signal using the ChemiDoc touch imaging system (Bio-Rad, Hercules, CA, USA).

### Immunohistochemical analysis

The sections used for the immunohistochemical analysis were collected from the patients who underwent gastrectomy at the Korea University Ansan Hospital and Seoul National University Hospital. In total, 42 normal gastric tissue, 130 EGC tissue, and 255 AGC tissue. The sections were deparaffinized in xylene and dehydrated in graded ethanol series, followed by heat-induced epitope retrieval in bond epitope retrieval solution. HOXC6 protein expression was detected using a primary antibody against HOXC6 (anti-HOXC6 antibody produced in rabbit, 1:50, OASG03595, Aviva, USA). After incubation with a bond polymer refine and 3,3′-diaminobenzidine (DAB) detection kits, the slides were rinsed and counterstained with Harris hematoxylin. The presence of staining in the nucleus, and not in the cytoplasm, was considered a positive result. The use of tissue samples was approved by the Institutional Review Board of Ansan Medical center (IRB no: 2018AS0092) and Seoul National University Hospital (IRB no: H-0808-064-254).

### Statistical analysis

All statistical analyses were performed with the SPSS 20.0 software (SPSS, Chicago, IL, United States). The Mann-Whitney U and Kruskal-Wallis tests were utilized to evaluate the relationship between the clinicopathologic parameters and the level of mRNA expression. The statistical significance of the western blot data was analyzed by Student's t-test using GraphPad PRISM version 5.0 (GraphPad Software, San Diego, CA, USA). The chi-square test for trend, and chi-square test were used to evaluate the relationship between the clinicopathologic parameters and protein expression. Survival was estimated using Kaplan-Meier analysis.

## Results

### Identifying the molecular signature of gastric cancer

Through the RNA sequencing analysis, we identified 20 genes with over 2-fold changes in the mRNA expression with progression. Among these genes, there was a steady rise in the expression level of two genes from normal tissue, to AGC without lymph node metastases, and finally, to AGC with lymph node metastases. In contrast, 18 genes were progressively decreased (Tables 1 and 2). Of these genes, significant difference was found only in the expression of *HOXC6* out of all the upregulated DEGs and *IGFBP6* out of all the downregulated DEGs (p<0.05). Hence, we decided to focus on the homeobox gene, *HOXC6*.

**Table 1:** Differentially expressed genes from RNA sequencing.

Expression	Gene symbol	Description	HGNC ID	chromosome
**Up**	HOXC6	Homeobox C6	5128	chr12
	CEMIP	Cell migration inducing hyaluronidase 1	29213	chr15
**Down**	DPT	Dermatopontin	3011	chr1
	ANGPTL1	Angiopoietin-like 1	489	chr1
	LDB3	LIM domain binding 3	15710	chr10
	LYVE1	Lymphatic vessel endothelial hyaluronan receptor 1	14687	chr11
	IGFBP6	Insulin-like growth factor binding protein 6	5475	chr12
	CFD	Complement factor D	2771	chr19
	KCNK3	Potassium two pore domain channel subfamily K member 3	6278	chr2
	SCN7A	Sodium voltage-gated channel alpha subunit 7	10594	chr2
	KCNE2	Potassium voltage-gated channel subfamily E regulatory subunit 2	6242	chr21
	CLEC3B	C-type lectin domain family 3 member B	11891	chr3
	A4GNT	Alpha-1,4-N-acetylglucosaminyltransferase	17968	chr3
	KCNMB2	Potassium calcium-activated channel subfamily M regulatory beta subunit 2	6286	chr3
	ADH1B	Alcohol dehydrogenase 1B (class I), beta polypeptide	250	chr4
	LIFR	LIF receptor subunit alpha	6597	chr5
	NPY	Neuropeptide Y	7955	chr7
	NEFM	Neurofilament medium	7734	chr8
	PGM5-AS1	PGM5 antisense RNA 1	44181	chr9
	PLP1	Proteolipid protein 1	9086	chrX

HGNC ID, HUGO Gene Nomenclature Committee ID

**Table 2:** Level of fold change in differentially expressed genes from RNA sequencing.

	Fold change		p-value	
Gene symbol	AGC node -/ Control	AGC node +/ AGC node -	AGC node -/ Control	AGC node +/ AGC node -
**HOXC6**	16.252	2.946	0.002	0.004
CEMIP	8.037	2.081	0.004	0.156
DPT	0.138	0.367	0.000	0.277
ANGPTL1	0.213	0.493	0.014	0.285
LDB3	0.316	0.364	0.010	0.118
LYVE1	0.174	0.457	0.002	0.400
**IGFBP6**	0.462	0.427	0.005	0.003
CFD	0.283	0.311	0.028	0.266
KCNK3	0.272	0.447	0.026	0.091
SCN7A	0.275	0.229	0.038	0.195
KCNE2	0.156	0.485	0.015	0.600
CLEC3B	0.333	0.424	0.012	0.264
A4GNT	0.218	0.500	0.045	0.388
KCNMB2	0.309	0.212	0.031	0.097
ADH1B	0.206	0.317	0.029	0.362
LIFR	0.401	0.417	0.006	0.059
NPY	0.086	0.374	0.001	0.170
NEFM	0.271	0.191	0.017	0.182
PGM5-AS1	0.335	0.136	0.037	0.213
PLP1	0.156	0.309	0.027	0.190

AGC node-, Advanced gastric cancer without lymph node metastasis; AGC node +, Advanced gastric cancer with lymph node metastasis; Control, Matched normal gastric tissue

To further investigate the significance of *HOXC6*, we used the Oncomine online database to evaluate the relative expression on available datasets. Remarkably, Oncomine dataset showed upregulation of *HOXC6* mRNA in the GC compared to other cancer types. Significantly increased expression of *HOXC6* mRNA in GC / Control was also confirmed (p = 6.75E-11). *HOXC6* mRNA ranked in the top third percentile of 19,574 genes. The results of the analysis are shown in S1 Fig.

**Fig 1:**
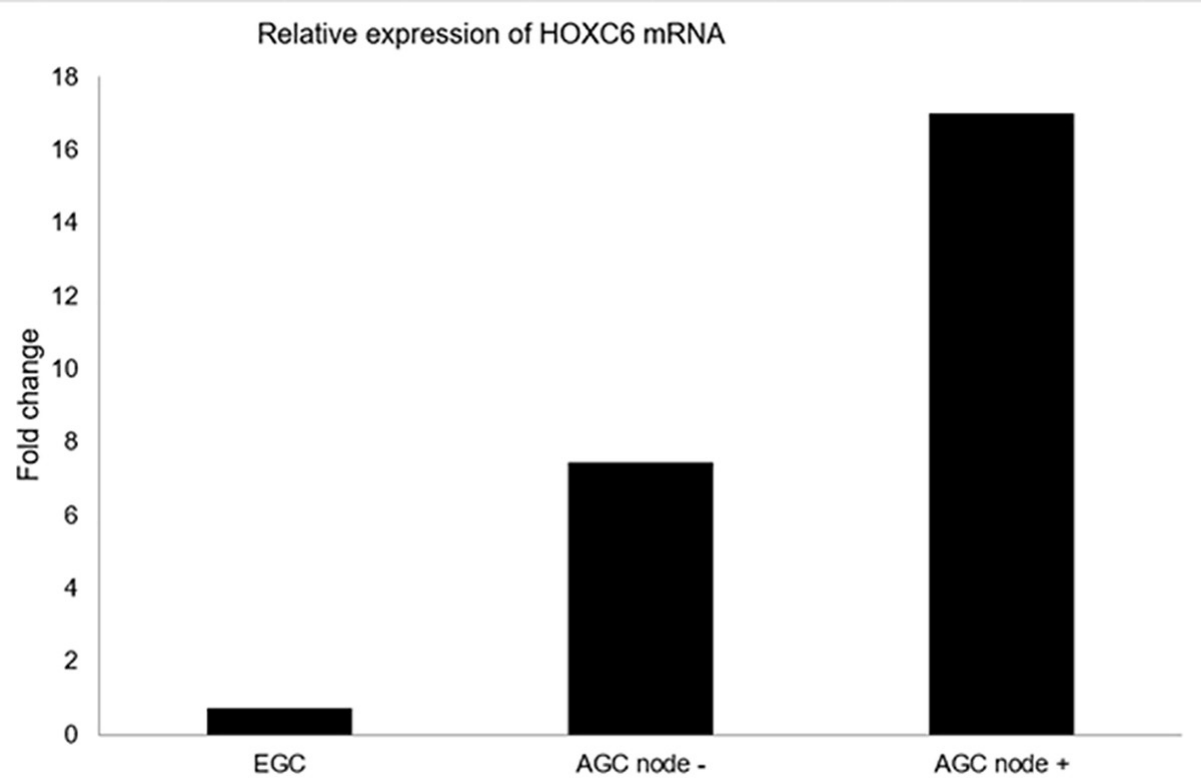
Analysis of *HOXC6* mRNA level using qRT-PCR.

To further validate the result of RNA sequencing, we performed real time PCR on normal gastric tissue, EGC tissue, AGC without lymph node metastasis, and AGC with lymph node metastasis. As shown in Fig 1, there were 7.45-fold and 16.98-fold increases in the *HOXC6* mRNA level in the AGC tissues without lymph node metastasis and with lymph node metastasis, respectively, compared to that observed in the matched normal gastric tissue. However, the level of *HOXC6* mRNA in the EGC tissues were lower, only 0.71-fold, than that in the matched normal gastric tissue.

### Correlation between *HOXC6* RNA *in*-*situ* hybridization results and clinicopathologic parameters

**Fig 2:**
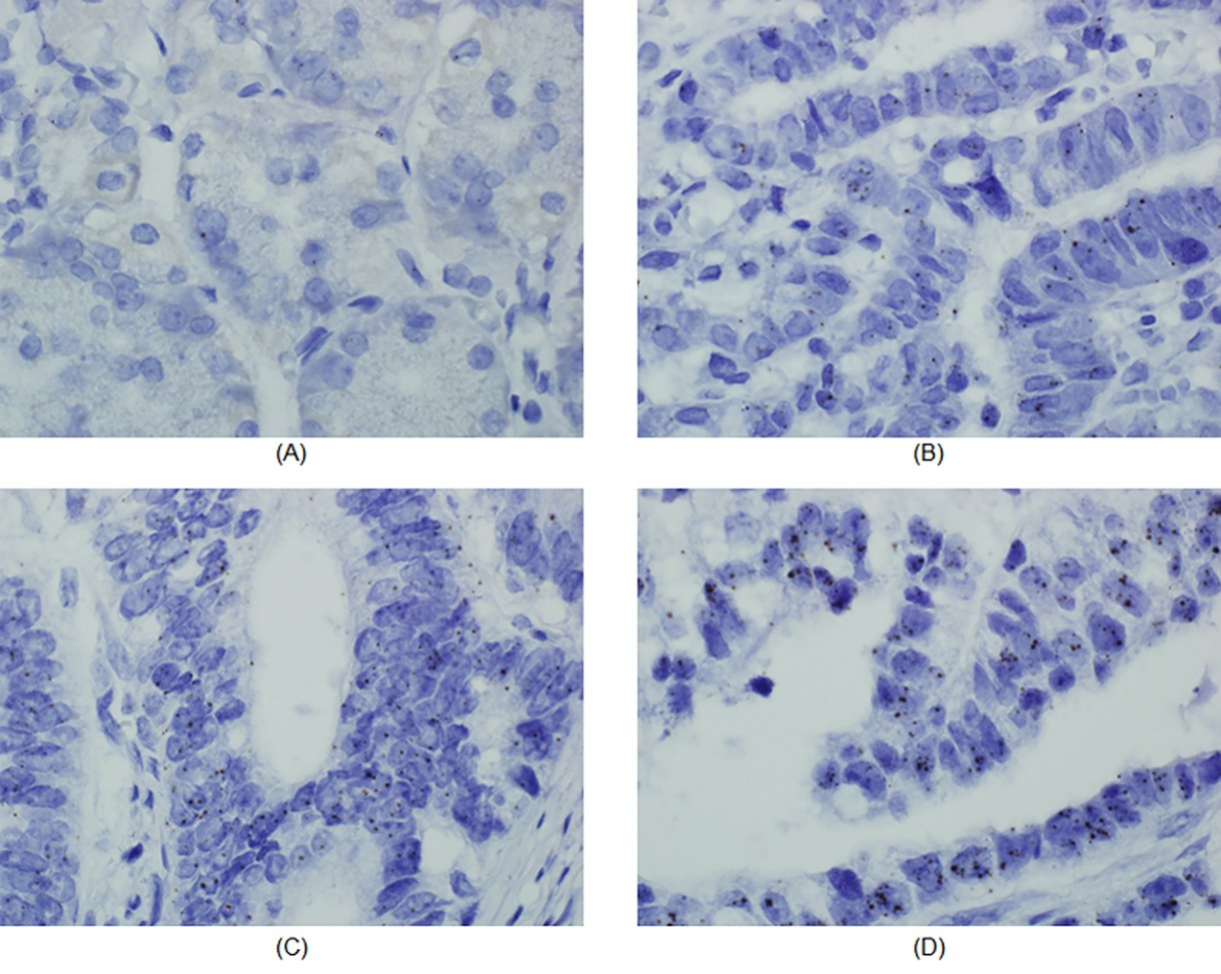
*HOXC6* (RNA *in*-*situ* hybridization) expression. (A) Control (B) EGC (C) AGC without metastasis (D) AGC with metastasis. At 1000x magnification.

The characteristics of the patients and expression counts are listed in Table 3 and Fig 2. The expression levels of *HOXC6* mRNA in GC were significantly higher than those in the normal gastric tissues (p<0.0001, Mann-Whitney U test). Additionally, after the patients were classified into groups, as control, EGC, and AGC based on the comparative analysis, the results showed that there was a gradual increase in the levels of *HOXC6* mRNA levels with cancer progression (p<0.0001, Kruskal-Wallis test). The difference was also statistically significant in the four groups, namely the control, EGC, AGC without lymph node metastasis, and AGC with lymph node metastasis. The data revealed that expression level of *HOXC6* mRNA was higher in GC at advanced stages (P<0.05, Kruskal-Wallis test). Although it was not statistically significant, the group of patients with lymph node metastasis showed higher expression counts compared to the group without metastasis. Moreover, the *HOXC6* mRNA levels in the patients aged 60 and above were significantly higher than those in the patients aged less than 60. Other than those, we did not observe any direct effects of factors such as gender, differentiation, size, and location in *HOXC6* mRNA expression (p>0.05).

**Table 3:** Relationship between expression counts by RNA *in-situ* hybridization with clinicopathologic characteristics.

Factor	N	Mean (count/100 cells)	SD	p-value
**Total**	51			
**Group**				**<0.001**^a^
Control	8	4.88	3.72	
Early gastric cancer	15	69.67	18.34	
Advanced gastric cancer	28	79.25	42.66	
**Gender**				0.602^b^
Male	39	66.95	41.17	
Female	12	57.67	46.39	
**Age**				**0.015**^b^
<60	25	50.88	44.45	
≥60	26	78.12	35.78	
**Differentiation**				0.294^a^
Well	9	69.89	31.80	
Intermediate	24	83.50	37.49	
Poor	10	63.10	34.92	
**Tumor Size** (in Diameter)				0.616^b^
<5 cm	24	78.13	33.18	
≥5 cm	19	73.11	40.24	
**Location**				0.363^b^
Cardiac	5	99.60	49.99	
Non-cardiac	38	72.79	33.52	
**Primary tumor depth (T)**				0.243^a^
T1	15	69.67	18.34	
T2	7	58.86	50.43	
T3	17	82.88	38.39	
T4	4	99.50	42.92	
**Nodal status (N)**				0.384^b^
N0	26	71.04	31.47	
N1~3	17	83.35	42.15	
**Distant metastasis (M)**				0.532^b^
M0	36	74.25	36.87	
M1	7	84.43	33.09	
**Stage**				**0.008**^a^
Ⅰ	18	57.94	25.35	
Ⅱ	10	103.80	26.48	
Ⅲ	8	74.00	49.00	
Ⅳ	7	84.43	33.09	

^a^ Kruskal-Wallis test

^b^ Mann-Whitney U test

### Clinicopathologic significance of *HOXC6* mRNA expression in gastric cancer patients

**Fig 3:**
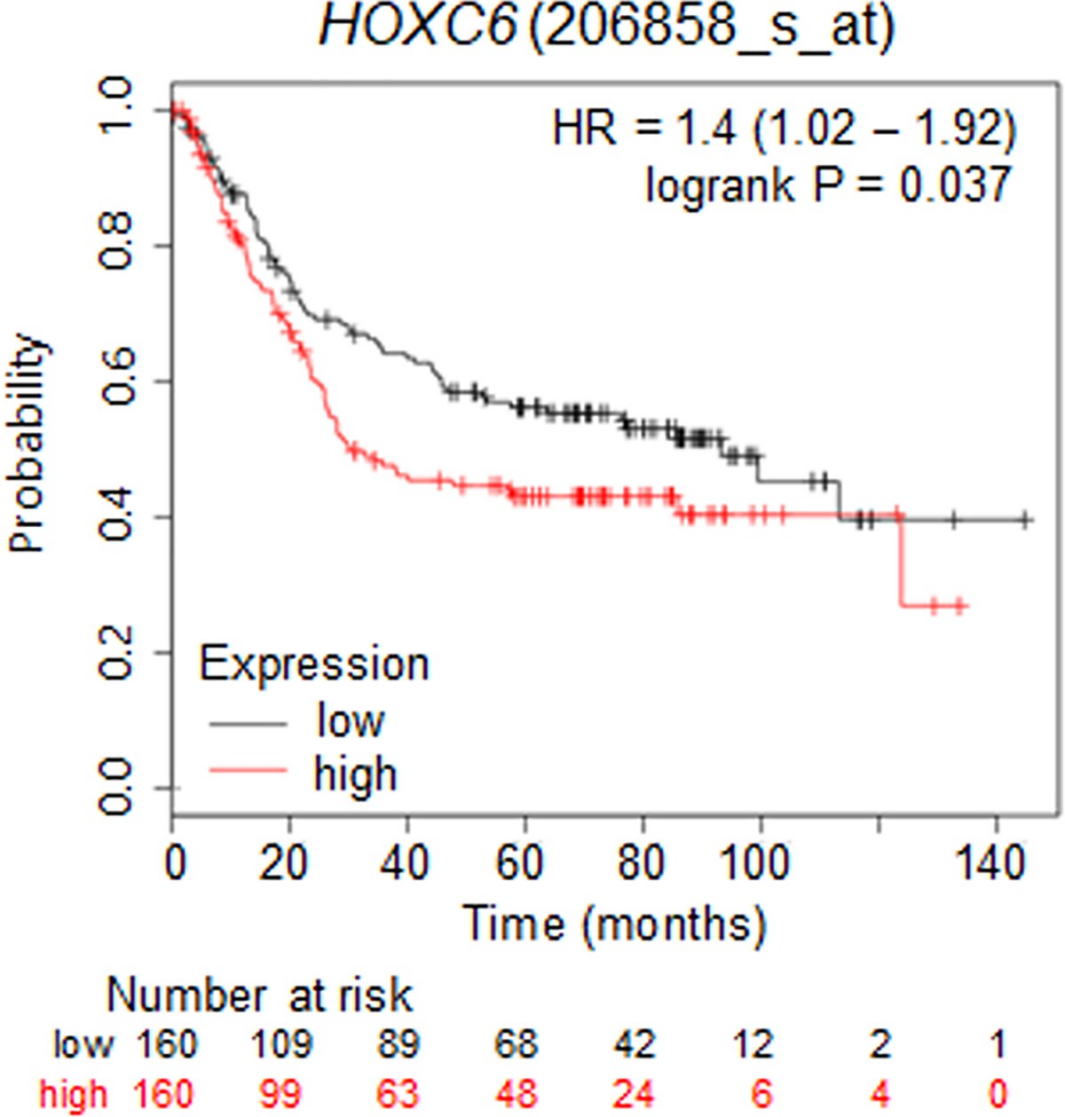
High *HOXC6* expression in gastric cancer tissues indicated poor overall survival outcome. Kaplan-Meier survival curve with *HOXC6* expression in tumor tissues for GC patients. HR, hazard ratio.

Kaplan-Meier curves showed that patients with higher *HOXC6* mRNA expression had a lower overall survival rate than those in the group with lower *HOXC6* mRNA expression (p<0.05) (Fig 3). The median survival time of GC patients with high *HOXC6* mRNA expression was 30.2 months, which was shorter than that of GC patients with low *HOXC6* mRNA expression (median 93.2 months). In summary, high expression of *HOXC6* mRNA is significantly associated with poor clinical prognosis

### Western blot analysis

**Fig 4:**
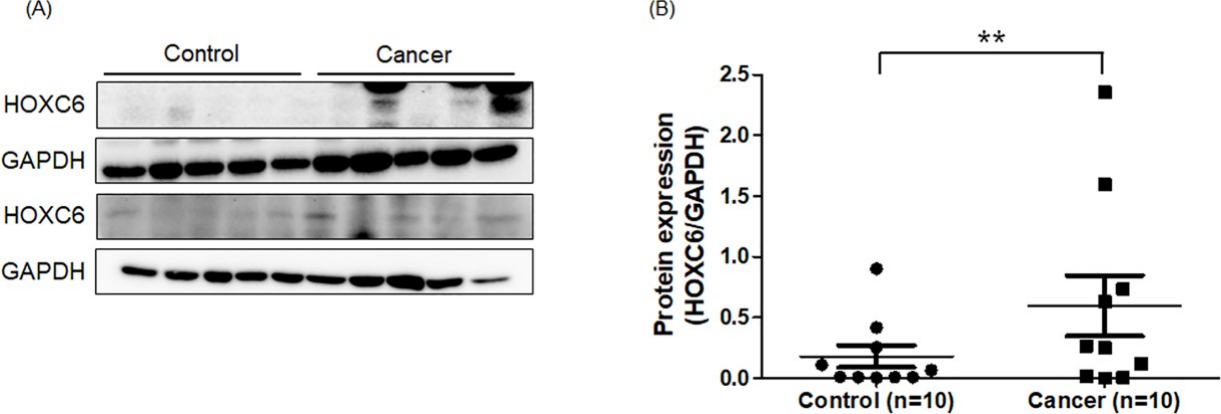
Western blot analysis for HOXC6 protein in normal gastric tissue and gastric cancer. The expression levels of HOXC6 protein in ten representative paired samples by western blot analysis. (A) HOXC6 protein expression was relatively higher in GC tissues compared with normal tissues. GAPDH was used as the endogenous control. (B) Results of relative quantification of the intensity of HOXC6 bands from GC tissues and normal controls. p<0.05 was considered statistically significant. **p<0.05 based on the Student’s t test. Values are mean ± SEM.

A western blot trial was performed to further confirm the HOXC6 protein expression. The results revealed that GC tissues exhibited higher expression of HOXC6 protein compared to corresponding normal controls significantly. The result of ten paired representative samples is shown in Fig 4 and S2 Fig.

### Immunohistochemical analysis of HOXC6 in gastric cancer

According to the immunohistochemical results, HOXC6 protein was mainly expressed in the nucleus and the cytoplasm. In 45.2% of the total number of cases, there was detectable nuclear staining pattern. Among these tissues, only 28.57% of the corresponding normal tissues showed positive staining. In comparison, expression of HOXC6 was observed in 181 (47.01%) of the tumor cases. There was a statistically significant difference between controls and cancers (p = 0.023). Moreover, the HOXC6 expression in the patients aged 60 and above were significantly higher than those in the patients aged less than 60, consistent with RNA *in*-*situ* hybridization. Male GC had a higher proportion of HOXC6 positivity than female GC. But otherwise, there was no statistically significant difference based on size, tumor depth (T), stage and nodal status (N) contrary to our expectation (Table 4 and S3 Fig).

**Table 4:** Relationship between HOXC6 protein expression and clinicopathologic characteristics.

Parameters	N	Positive (%)	p-value
**Total**	427	193 (45.2%)	**0.023**^a^
Control	42	12 (28.57%)	
Gastric cancer	385	181 (47.01%)	
**Group**			0.088^b^
Control	42	12 (28.57%)	
Early gastric cancer	130	61 (46.92%)	
Advanced gastric cancer	255	120 (47.06%)	
**Gender**			**<0.01**^a^
Male	263	140 (53.23%)	
Female	122	41 (33.61%)	
**Age**			**0.044**^a^
<60	189	79 (41.80%)	
≥60	196	102 (52.04%)	
**Tumor Size**			0.263^a^
<5 cm	220	98 (44.55%)	
≥5 cm	165	83 (50.30%)	
**Primary tumor depth (T)**			0.624^b^
T1	130	61 (46.92%)	
T2	75	31 (41.33%)	
T3	157	78 (49.68%)	
T4	23	11 (47.83%)	
**Nodal status (N)**			0.644^a^
N0	192	88 (45.83%)	
N1~3	193	93 (48.19%)	
**Stage**			0.914^b^
Ⅰ	170	78 (45.88%)	
Ⅱ/Ⅲ	191	93 (48.69%)	
Ⅳ	24	10 (41.67%)	

^a^ Chi-square test

^b^ Chi-square test for trend

## Discussion

In the current study, we attempted to screen for DEGs and validated the potential biomarker genes in GC using multiple methods. Using these methods, we identified a gene, *HOXC6* that was significantly upregulated in GC tissues compared with normal controls. Concomitantly, *HOXC6* mRNA overexpression was found to be associated with poor prognostic factors in GC patients.

The homeobox (HOX) gene family is a crucial regulatory factor in growth and differentiation. In humans, an estimated 257 genes have been identified, and the most of them are dispersed throughout the entire genome [13, 14]. HOX genes belong to a family of 39 transcription factors that are divided into four clusters, HOXA, HOXB, HOXC, and HOXD, which can be mapped onto the chromosome 7p15, 17q21.2, 12q13, and 2q31 loci, respectively [15]. In different forms of cancer, homeobox genes are deregulated and act as tumor modulators [16]. Some studies that have been published on HOXC6 so far, particularly in prostate cancer [17, 18]. These studies have not only revealed that HOXC6 is overexpressed in prostate cancer but have also identified several of its targets. HOXC6 directly regulates the expression of bone morphogenic protein 7 (BMP-7), fibroblast growth factor receptor 2 (FGFR-2), insulin-like growth factor binding protein 3 (IGFBP-3), and platelet-derived growth factor receptor alpha (PDGFR-α) in prostate cells and indirectly influences the Notch and Wnt signaling pathways [17]. It has also been revealed that HOXC6 can help in preventing apoptosis of prostate cancer cells by repressing the expression of neutral endopeptidase (NEP) and IGFBP-3 [18]. However, HOX genes are known to function distinctively depending on the expressed tissue [16], there are limited studies available on *HOXC6* in GC. The most notable studies in GC were conducted by Chen et al [19, 20] who reported *HOXC6* was highly expressed in GC clinical specimens and investigated the effect of *HOXC6* on the expression of matrix metalloproteinase (MMP) family genes in vitro. The study suggested a possible mechanism by which *HOXC6* positively regulated *MMP9* via extracellular-signal-regulated kinase (ERK) activation. The most recent study explored isoforms of HOXC6 and found that only HOXC6-2 isoform serves a primary carcinogeneic role in GC [21]. The aforementioned studies selected *HOXC6* as a candidate predictor in GC since *HOXC6* documented in other cancers [17, 22, 23]. Although they compared *HOXC6* expression in cancer tissues with that in neighboring normal tissues, the potentially crucial candidate gene selection was not based on actual screening methods such as sequencing.

In this study, we screened the differentially expressed gene *HOXC6* from a large number of genes through RNA sequencing. Despite the small numbers, we thought that it signifies actual examining differentially expressed genes among Korean patients. To establish reliability, we utilized Oncomine online database to confirm the expression of *HOXC6*. The present study revealed clinically significant finding especially *in-situ* hybridization. *In*-*situ* hybridization is a useful diagnostic tool to examine gene expression in the pathology laboratory along with immunohistochemistry. Whereas immunohistochemistry often shows false-positive stain due to immunoglobulin and intracellular proteins, *in*-*situ* hybridization does not cause the problem because RNAs are mostly intracellular and *in*-*situ* hybridization detects de novo gene products [24]. It is useful to detect the biomarker simultaneously with cancer diagnosis under a brightfield light microscopy and easy to analyze and interpret visually. No aforementioned studies have done this method to detect *HOXC6* in GC, utilizing this method could give analytic advantages. In addition, survival analysis using Kaplan-Meier plotter, we included more patients, compared to previous studies to get more credible results.

These results indicated that *HOXC6* mRNA expression might be linked with poor clinical prognosis and are consistent with previous studies, which were conducted in GC and other malignant tumors such as prostate cancer, esophageal cancer and hepatocellular carcinoma [20, 22, 25, 26]. Therefore, we conclude that *HOXC6* might be a potential biomarker for predicting prognosis.

The genes that are expressed in cancers, particularly the ones that encode proteins involved in the oncogenic process, might act as ideal diagnostic or therapeutic biomarkers. In fact, established results showing upregulation of HER2 enabled the treatment of HER2-positive GC patients [27]. Similarly, with increasing understanding of gene expression changes, we can improve the availability of targets for cancer therapy.

There are some limitations in the present study. First of all, although we have validated the expression and prognostic significance of *HOXC6* using various methods, the specific function and molecular mechanism of *HOXC6* in GC need to be further explored. Another one is that our findings were not strongly supported by the immunohistochemistry results. Although the HOXC6 protein expression levels were increased in GC tissues compared to the matched normal tissues, the results of relationship between tumor invasion depth and HOXC6 protein expression obtained were not statistically significant unlike HOXC6 mRNA expression. Generally, it is difficult to find suitable antibodies for immunohistochemical analysis. Moreover, it is hard to determine an optimal cut-off value for immunohistochemical staining, which might also be the cause. For ease in using HOXC6 antibody efficaciously, regulatory developments, adequate antibody selection among the various manufacturers, staining optimization, and test validation should be addressed.

In summary, this study suggested that *HOXC6* mRNA might act as a potential candidate diagnostic and prognostic biomarker in individual GC patients. Our findings might also serve as the foundation for developing novel therapeutic strategies.

## Supporting information

S1 Fig (A) Box plot comparing *HOXC6* mRNA expression in normal gastric tissues (left plot) and gastric cancer tissues (right plot) was derived from the Oncomine database. The fold change of *HOXC6* mRNA was analyzed by the Oncomine database. The data are gastric intestinal adenocarcinoma tissues (GC) relative to normal gastric tissues (Control). (B) Box plot comparing *HOXC6* mRNA expression between the cancers. BC, Breast cancer; CC, Colon cancer; GC, Gastric cancer; PC, Pancreatic cancer.
(TIF)

S2 Fig Original microphotographs for HOXC6 protein expression by western blot analysis in gastric cancer tissues and normal gastric tissues. Molecular weight of HOXC6 isoform is 27 kDa. (A) HOXC6 (B) GAPDH.
(PDF)

S3 Fig Representative microphotographs for HOXC6 protein expression by immunohistochemical staining in gastric cancer tissues and normal gastric tissues. (A) Control (B) Gastric cancer. At 1000x magnification.
(TIF)

## References

[1] Bray F, Ferlay J, Soerjomataram I, Siegel RL, Torre LA, Jemal A. Global cancer statistics 2018: GLOBOCAN estimates of incidence and mortality worldwide for 36 cancers in 185 countries. CA: A Cancer Journal for Clinicians. 2018;68(6):394–424. 30207593 10.3322/caac.21492

[2] Kim YG, Kong S-H, Oh S-Y, Lee K-G, Suh Y-S, Yang J-Y, et al Effects of screening on gastric cancer management: comparative analysis of the results in 2006 and in 2011. J Gastric Cancer. 2014;14(2):129–34. 10.5230/jgc.2014.14.2.129 25061541 PMC4105378

[3] Kim SG, Park CM, Lee NR, Kim J, Lyu DH, Park SH, et al Long-Term Clinical Outcomes of Endoscopic Submucosal Dissection in Patients with Early Gastric Cancer: A Prospective Multicenter Cohort Study. Gut Liver. 2018;12(4):402–10. 10.5009/gnl17414 29588436 PMC6027839

[4] Kusano T, Shiraishi N, Shiroshita H, Etoh T, Inomata M, Kitano S. Poor Prognosis of Advanced Gastric Cancer with Metastatic Suprapancreatic Lymph Nodes. Annals of Surgical Oncology. 2013;20(7):2290–5. 10.1245/s10434-012-2839-8 23299769 PMC3675275

[5] Gullo I, Carneiro F, Oliveira C, Almeida GM. Heterogeneity in Gastric Cancer: From Pure Morphology to Molecular Classifications. Pathobiology. 2018;85(1–2):50–63. 10.1159/000473881 28618420

[6] Wang Y. Identifying key stage-specific genes and transcription factors for gastric cancer based on RNA-sequencing data. Medicine. 2017;96(4):e5691 10.1097/MD.0000000000005691 28121923 PMC5287947

[7] Langmead B, Salzberg SL. Fast gapped-read alignment with Bowtie 2. Nature methods. 2012;9(4):357–9. 10.1038/nmeth.1923 22388286 PMC3322381

[8] Quinlan AR, Hall IM. BEDTools: a flexible suite of utilities for comparing genomic features. Bioinformatics (Oxford, England). 2010;26(6):841–2. 10.1093/bioinformatics/btq033 PMC2832824 20110278

[9] Gentleman RC, Carey VJ, Bates DM, Bolstad B, Dettling M, Dudoit S, et al Bioconductor: open software development for computational biology and bioinformatics. Genome biology. 2004;5(10):R80 10.1186/gb-2004-5-10-r80 15461798 PMC545600

[10] Rhodes DR, Yu J, Shanker K, Deshpande N, Varambally R, Ghosh D, et al ONCOMINE: a cancer microarray database and integrated data-mining platform. Neoplasia (New York, NY). 2004;6(1):1–6. 10.1016/s1476-5586(04)80047-2 PMC1635162 15068665

[11] D'Errico M, de Rinaldis E, Blasi MF, Viti V, Falchetti M, Calcagnile A, et al Genome-wide expression profile of sporadic gastric cancers with microsatellite instability. European journal of cancer (Oxford, England: 1990). 2009;45(3):461–9. 10.1016/j.ejca.2008.10.032 19081245

[12] Wang F, Flanagan J, Su N, Wang LC, Bui S, Nielson A, et al RNAscope: a novel in situ RNA analysis platform for formalin-fixed, paraffin-embedded tissues. The Journal of molecular diagnostics: JMD. 2012;14(1):22–9. 10.1016/j.jmoldx.2011.08.002 22166544 PMC3338343

[13] Rodrigues MF, Esteves CM, Xavier FC, Nunes FD. Methylation status of homeobox genes in common human cancers. Genomics. 2016;108(5–6):185–93. 10.1016/j.ygeno.2016.11.001 27826049

[14] Luo Z, Rhie SK, Farnham PJ. The Enigmatic HOX Genes: Can We Crack Their Code? Cancers (Basel). 2019;11(3):323. 10.3390/cancers11030323 PMC6468460 30866492

[15] Ruddle FH, Bartels JL, Bentley KL, Kappen C, Murtha MT, Pendleton JW. Evolution of Hox genes. Annual review of genetics. 1994;28:423–42. 10.1146/annurev.ge.28.120194.002231 7893134

[16] Abate-Shen C. Deregulated homeobox gene expression in cancer: cause or consequence? Nature reviews Cancer. 2002;2(10):777–85. 10.1038/nrc907 12360280

[17] McCabe CD, Spyropoulos DD, Martin D, Moreno CS. Genome-wide analysis of the homeobox C6 transcriptional network in prostate cancer. Cancer research. 2008;68(6):1988–96. 10.1158/0008-5472.CAN-07-5843 18339881 PMC2584783

[18] Ramachandran S, Liu P, Young AN, Yin-Goen Q, Lim SD, Laycock N, et al Loss of HOXC6 expression induces apoptosis in prostate cancer cells. Oncogene. 2005;24(1):188–98. 10.1038/sj.onc.1207906 15637592

[19] Chen SW, Zhang Q, Xu ZF, Wang HP, Shi Y, Xu F, et al HOXC6 promotes gastric cancer cell invasion by upregulating the expression of MMP9. Molecular medicine reports. 2016;14(4):3261–8. 10.3892/mmr.2016.5640 27573865

[20] Zhang Q, Jin XS, Yang ZY, Wei M, Liu BY, Gu QL. Upregulated Hoxc6 expression is associated with poor survival in gastric cancer patients. Neoplasma. 2013;60(4):439–45. 10.4149/neo_2013_057 23581417

[21] Lin J, He J, He X, Wang L, Xue M, Zhuo W, et al HoxC6 Functions as an Oncogene and Isoform HoxC6-2 May Play the Primary Role in Gastric Carcinogenesis. Digestive Diseases and Sciences. 2020. 10.1007/s10620-019-06013-7 31900716

[22] Du YB, Dong B, Shen LY, Yan WP, Dai L, Xiong HC, et al The survival predictive significance of HOXC6 and HOXC8 in esophageal squamous cell carcinoma. The Journal of surgical research. 2014;188(2):442–50. 10.1016/j.jss.2014.01.017 24525058

[23] Moon SM, Kim SA, Yoon JH, Ahn SG. HOXC6 is deregulated in human head and neck squamous cell carcinoma and modulates Bcl-2 expression. The Journal of biological chemistry. 2012;287(42):35678–88. 10.1074/jbc.M112.361675 22896703 PMC3471680

[24] Chu Y-H, Hardin H, Zhang R, Guo Z, Lloyd RV. In situ hybridization: Introduction to techniques, applications and pitfalls in the performance and interpretation of assays. Seminars in Diagnostic Pathology. 2019;36(5):336–41. 10.1053/j.semdp.2019.06.004 31227426

[25] Zhou J, Yang X, Song P, Wang H, Wang X. HOXC6 in the prognosis of prostate cancer. Artificial Cells, Nanomedicine, and Biotechnology. 2019;47(1):2715–20. 10.1080/21691401.2019.1635136 31271305

[26] Li PD, Chen P, Peng X, Ma C, Zhang WJ, Dai XF. HOXC6 predicts invasion and poor survival in hepatocellular carcinoma by driving epithelial-mesenchymal transition. Aging. 2018;10(1):115–30. 10.18632/aging.101363 29348394 PMC5811246

[27] Gravalos C, Jimeno A. HER2 in gastric cancer: a new prognostic factor and a novel therapeutic target. Annals of Oncology. 2008;19(9):1523–9. 10.1093/annonc/mdn169 18441328

