# Supplementary figures and images for: Increased HOXC6 mRNA expression is a novel biomarker of gastric cancer
Source: PLoS One. 2020 Aug 3;15(8):e0236811. doi: 10.1371/journal.pone.0236811 (PMC7398522; doi:10.1371/journal.pone.0236811)

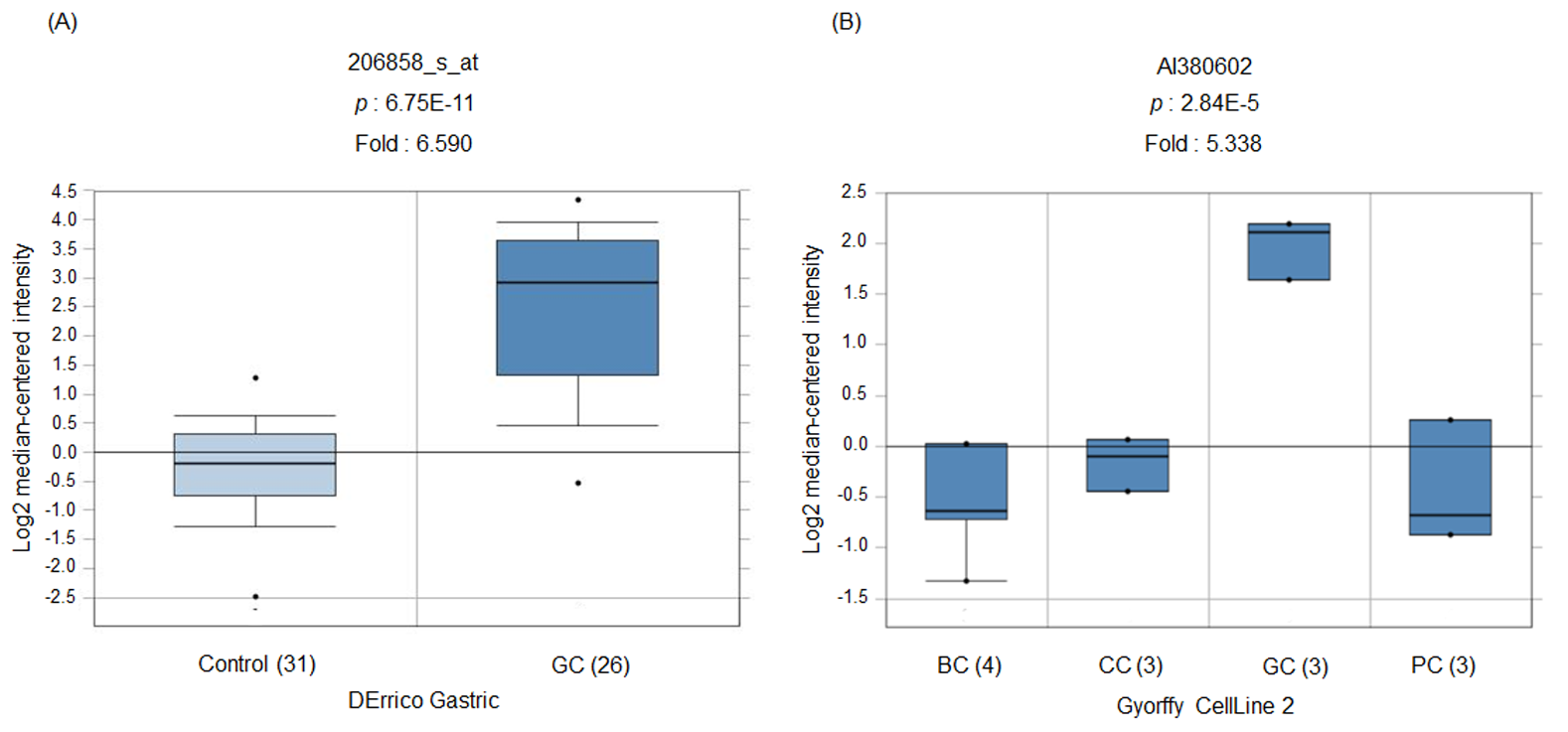

Supplement: S1 Fig — (A) Box plot comparing HOXC6 mRNA expression in normal gastric tissues (left plot) and gastric cancer tissues (right plot) was derived from the Oncomine database. The fold change of HOXC6 mRNA was analyzed by the Oncomine database. The data are gastric intestinal adenocarcinoma tissues (GC) relative to normal gastric tissues (Control). (B) Box plot comparing HOXC6 mRNA expression between the cancers. BC, Breast cancer; CC, Colon cancer; GC, Gastric cancer; PC, Pancreatic cancer. (TIF) [file pone.0236811.s001.tif]

**HOXC6**

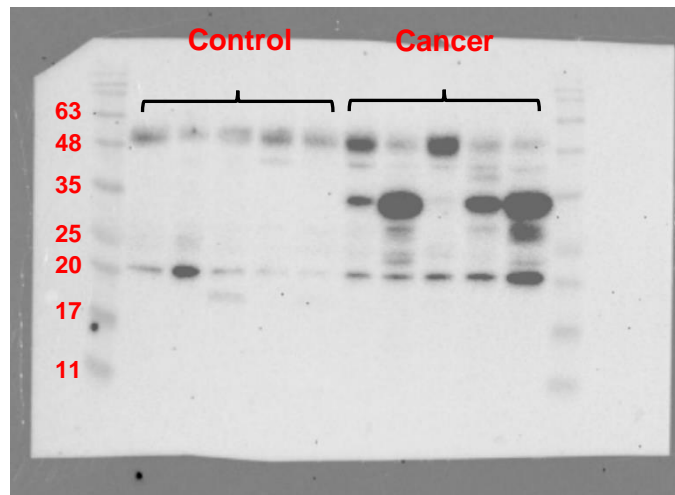

(A1)

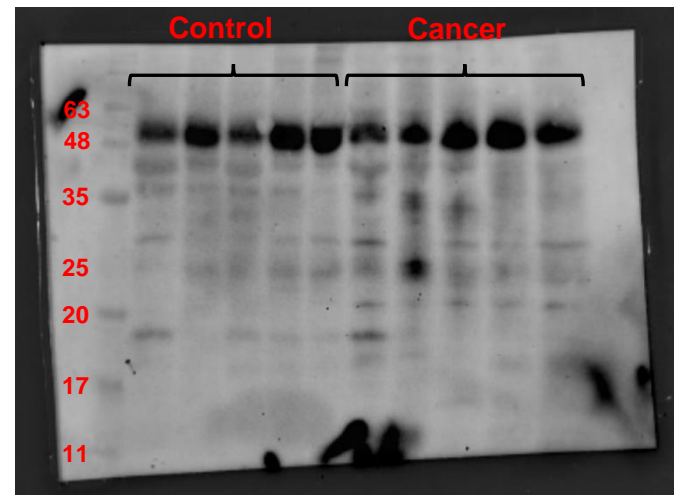

(A2)

**GAPDH**

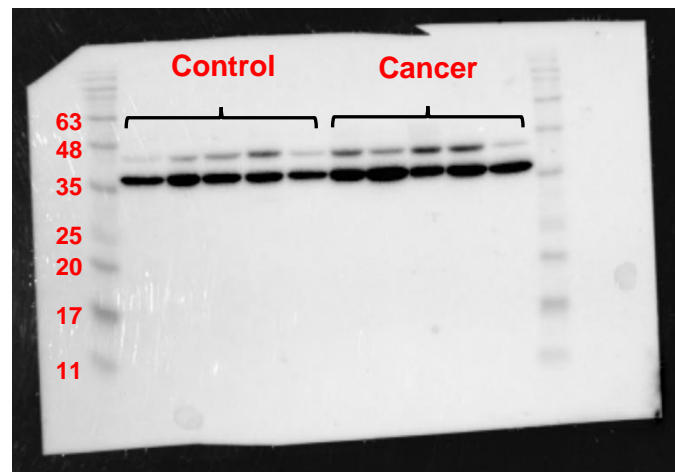

(B1)

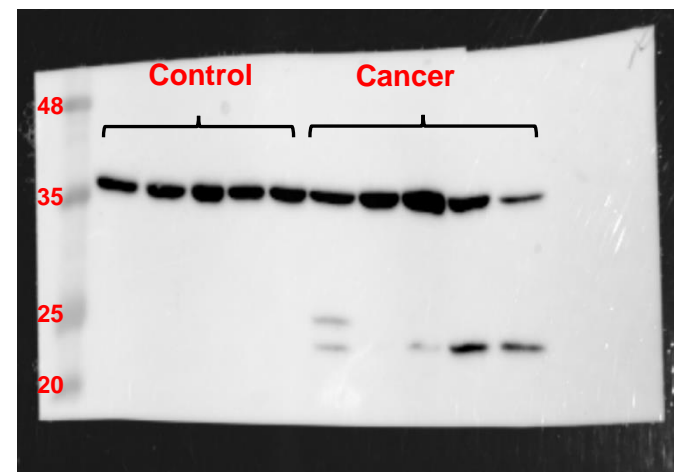

(B2)

Supplement: S2 Fig — Molecular weight of HOXC6 isoform is 27 kDa. (A) HOXC6 (B) GAPDH. (PDF) [file pone.0236811.s002.pdf]

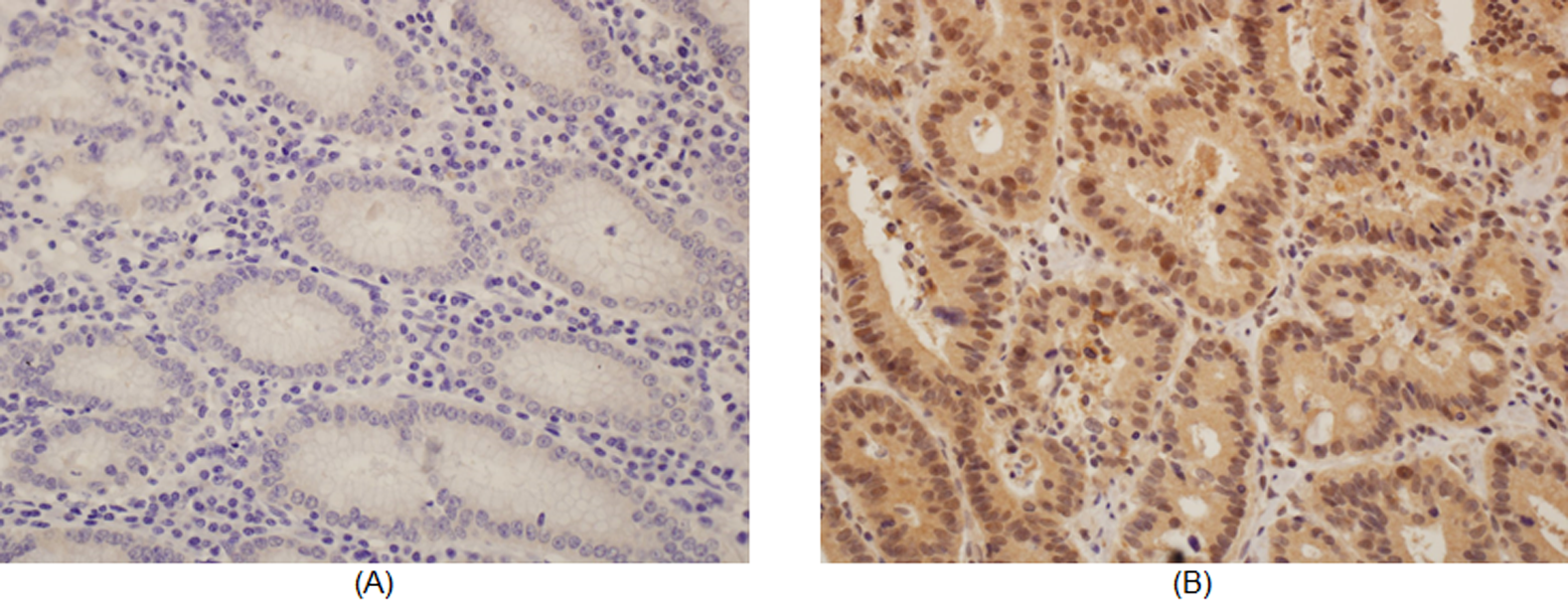

Supplement: S3 Fig — (A) Control (B) Gastric cancer. At 1000x magnification. (TIF) [file pone.0236811.s003.tif]
